# Processing of visual statistics of naturalistic videos in macaque visual areas V1 and V4

**DOI:** 10.1007/s00429-022-02468-z

**Published:** 2022-03-14

**Authors:** Gaku Hatanaka, Mikio Inagaki, Ryosuke F. Takeuchi, Shinji Nishimoto, Koji Ikezoe, Ichiro Fujita

**Affiliations:** 1 Graduate School of Frontier Biosciences, Osaka University, Suita, Osaka 565-0871 Japan; 2 Center for Information and Neural Networks, Osaka University and National Institute of Information and Communications Technology, Suita, Osaka 565-0871 Japan; 3 Faculty of Medicine, University of Yamanashi, Chuo, Yamanashi 409-3898 Japan

**Keywords:** Two-photon microscopy, Calcium imaging, Portilla–Simoncelli statistics, Encoding model analysis, Primary visual cortex, Functional architecture

## Abstract

Natural scenes are characterized by diverse image statistics, including various parameters of the luminance histogram, outputs of Gabor-like filters, and pairwise correlations between the filter outputs of different positions, orientations, and scales (Portilla–Simoncelli statistics). Some of these statistics capture the response properties of visual neurons. However, it remains unclear to what extent such statistics can explain neural responses to natural scenes and how neurons that are tuned to these statistics are distributed across the cortex. Using two-photon calcium imaging and an encoding-model approach, we addressed these issues in macaque visual areas V1 and V4. For each imaged neuron, we constructed an encoding model to mimic its responses to naturalistic videos. By extracting Portilla–Simoncelli statistics through outputs of both filters and filter correlations, and by computing an optimally weighted sum of these outputs, the model successfully reproduced responses in a subpopulation of neurons. We evaluated the selectivities of these neurons by quantifying the contributions of each statistic to visual responses. Neurons whose responses were mainly determined by Gabor-like filter outputs (low-level statistics) were abundant at most imaging sites in V1. In V4, the relative contribution of higher order statistics, such as cross-scale correlation, was increased. Preferred image statistics varied markedly across V4 sites, and the response similarity of two neurons at individual imaging sites gradually declined with increasing cortical distance. The results indicate that natural scene analysis progresses from V1 to V4, and neurons sharing preferred image statistics are locally clustered in V4.

## Introduction

The visual system performs complex analyses of input images derived from natural scenes. This complexity is highlighted by the fact that a wide variety of image statistics is necessary to synthesize artificial images that are perceptually indistinguishable from the original natural images (Freeman and Simoncelli 2011). A subset of the statistics can be extracted by Gabor-like filters, which analyze the spatial frequency and orientation of local regions of the image (spectral statistics). Another subset of statistics is derived from computations of the correlations across the outputs of pairs of Gabor-like filters with different positions, orientations, and scales (correlation statistics). Also important in determining the appearance of an image are the summary statistics of the luminance histogram (i.e., luminance distribution), such as the mean, variance, skewness, and kurtosis (marginal statistics; Motoyoshi et al. 2007). The ensemble of these image statistics (hereafter referred to as Portilla–Simoncelli statistics, or PS statistics) was first proposed for texture analysis/synthesis (Portilla and Simoncelli 2000), and its extension works well for explaining the perception of complex natural scenes by human observers (Freeman and Simoncelli 2011).

Simple cells in the primary visual cortex (V1) have a linear receptive field with a Gabor-like structure and are sensitive to the phase of grating stimuli (Hubel and Wiesel 1962; Jones and Palmer 1987a, b). Complex cells are tolerant to changes in the stimulus phase or stimulus position within their receptive field, and signal the local orientation, spatial frequency, motion, and binocular disparity independent of the sign of the stimulus contrast (Adelson and Bergen 1985; Watson and Ahumada 1985; Ohzawa et al. 1990). Spectral statistics are the major factors that explain the responses of both types of V1 neurons to texture images (Freeman et al. 2013). In contrast, correlation statistics are critical to eliciting responses to natural texture images in V2 (Freeman et al. 2013). In V4, which is a mid-tier stage along the ventral visual pathway, neuronal tuning to the correlation statistics and the skewness of the luminance histogram become more explicit than in V2 (Okazawa et al. 2017). Hierarchical processing along the ventral visual pathway appears to gradually create the representation of higher order features of texture images.

A challenge in analyzing neuronal tuning to PS statistics is the high dimensionality of the visual stimuli. Unlike spectral statistics, which simply represent the spatial frequency and orientation, correlation statistics consist of the combination of filter outputs across various positions, orientations, and scales, resulting in a large number of stimulus parameters. It is extremely difficult, if not impossible, to test all possible combinations of stimulus parameters in physiology experiments conducted in animals. Okazawa et al. (2015, 2017) mitigated this problem when analyzing the selectivity of V2 and V4 neurons for static texture stimuli by focusing on a subset of material surfaces and applying an adaptive sampling procedure (Yamane et al. 2008; Carlson et al. 2011). They successfully predicted the responses to texture images by means of a limited number of PS statistics. However, the contribution of marginal statistics to neuronal responses has been largely unexplored, because previous studies used stimuli with equalized luminance across stimulus images (Freeman et al. 2013; Okazawa et al. 2015, 2017). It remains unclear how well the responses to natural scenes can be characterized by PS statistics. Specifically, the luminance distribution is diverse across natural scene images, and therefore, there are significant changes in the marginal statistics of dynamic scenes such as those in a video. As a step to extend our understanding of neuronal processing of the image statistics in more general natural scenes, we examined the effects of the PS statistics in naturalistic videos on the neuronal responses in V1 and V4.

In primates, neurons in V1 and V4 are spatially clustered based on their specific functional properties. In both areas, neurons are primarily arranged according to their receptive field position, and organize the smooth retinotopic map (Hubel and Wiesel 1968; Desimone and Schein 1987; Gattass et al. 1988). Across the cortical surface of V1, neurons are also arranged according to their selectivity to orientation (Hubel and Wiesel 1968, 1977; Blasdel and Salama 1986) and spatial frequency (Nauhaus et al. 2012). In V4, neurons with preferences for color, binocular disparity, orientation, curvature, and non-Cartesian gratings tend to be clustered locally (Gallant et al. 1996; Watanabe et al. 2002; Tanabe et al. 2005; Kotake et al. 2009; Tanigawa et al. 2010; Hu et al. 2020; Tang et al. 2020; Srinath et al. 2021). These findings suggest that V4 is functionally heterogeneous across the cortical surface in terms of visual stimulus selectivities (Roe et al. 2012). The functional architecture underlying various PS statistics parameters beyond orientation and spatial frequency has not been systematically explored in any visual cortical area. Here, we studied the spatial distribution of neurons sensitive to different PS statistics in V1 and V4. For this purpose, we used in vivo two-photon calcium imaging to record activities from a large number of neurons, the locations of which can be determined with high spatial resolution. An adaptive sampling procedure cannot be combined with simultaneous recordings from many neurons, because it customizes the stimulus set through a genetic algorithm to suit the properties of a single target neuron. Instead, we applied an encoding-model approach (Gallant et al. 2011; Naselaris et al. 2011; Nishimoto et al. 2011) to characterize neuronal tuning to PS statistics. This approach enables analysis on visual properties of hundreds of neurons simultaneously recorded by two-photon calcium imaging (Ikezoe et al. 2018).

We showed that an encoding-model analysis of two-photon imaging data can capture responses of a subpopulation of neurons to natural scenes. Consistent with previous findings on orientation and spatial frequency preferences, we found that neurons whose responses were determined largely by low-level statistics (spectral statistics) were dominant at most imaging sites in V1. In V4, the relative contribution of marginal and correlation statistics became greater. Neurons sensitive to luminance distribution and/or higher order statistics such as linear scale correlation dominated many V4 sites, while some sites contained many neurons responding to low-level image statistics. Within a single V4 site, response similarity between two neurons was highest for the nearest neighbors, and gradually declined as the intercellular distance increased. These findings suggest that neurons processing different categories of image statistics are locally clustered in V4.

## Materials and methods

We performed two-photon calcium imaging in areas V1 and V4 of monkeys. All experimental and animal care procedures were approved by the animal experiment committee of Osaka University (permit numbers: FBS-12-017, FBS-18-005), and conformed to the *Guide for the Care and Use of Laboratory Animals* issued by the National Institutes of Health, USA (1996). The recording data for V1 largely overlapped with those presented in a previous study (Ikezoe et al. 2018), and here we subjected those data to novel analyses.

### Animal preparation

We used two adult female monkeys (*Macaca fascicularis*, 2.3 and 3.2 kg, 3 and 4 years, respectively). An initial aseptic surgery was performed for later repeat recordings (Ikezoe et al. 2013, 2018). Atropine sulfate (Mitsubishi Tanabe Pharma, Osaka; 0.025 mg/kg, intramuscular) was administered prior to each day’s recording sessions. Then, anesthesia was induced with ketamine hydrochloride (Daiichi-Sankyo, Tokyo; 11.5 mg/kg, intramuscular), and maintained with isoflurane (1–3%) in a mixture of nitrous oxide and oxygen (7:3). We performed a small craniotomy and durotomy (2–3 mm in diameter) over the region of V1 or V4 to be imaged. The exposed dura and cortex were covered with 2% agar and a 0.13–0.17-mm-thick cover glass (see Fig. 1 of Ikezoe et al. 2013). After this surgical procedure, we switched from isoflurane to fentanyl citrate (Daiichi-Sankyo; 10 µg/kg/h, intravenous) for analgesia (Popilskis and Kohn 1997). During imaging, vecuronium bromide (Merck Sharp and Dohme, Tokyo; 0.08 mg/kg/h, intravenous) was given to the monkeys to prevent eye movements, and they were artificially ventilated with a mixture of nitrogen and oxygen. We maintained the body temperature at 37–38 °C, and end-tidal CO_2_ at 4.0–5.5%. An electrocardiogram, blood pressure, and arterial oxygen-saturation levels were continuously monitored and maintained within appropriate ranges. Phenylephrine hydrochloride and tropicamide were applied to the eyes to relax accommodation and dilate the pupils. Corneas were covered with contact lenses of appropriate curvature, power, and pupil diameter (3 mm) to prevent drying and to allow the images on the stimulus display to be focused on the retina (Tamura et al. 2004). At the end of each recording session, the monkeys were administered neostigmine methylsulfate (Shionogi, Osaka; 0.1 mg/kg, intramuscular) to aid in recovering spontaneous respiration, as well as ketoprofen (Nissin Pharmaceutical, Yamagata, 0.8 mg/kg, intramuscular) for post-surgery analgesia. They were then returned to their cages and given ketoprofen and antibiotics (piperacillin sodium, Taisho Toyama Pharmaceutical, Tokyo) for 1 week after the experiment.

**Fig. 1:**
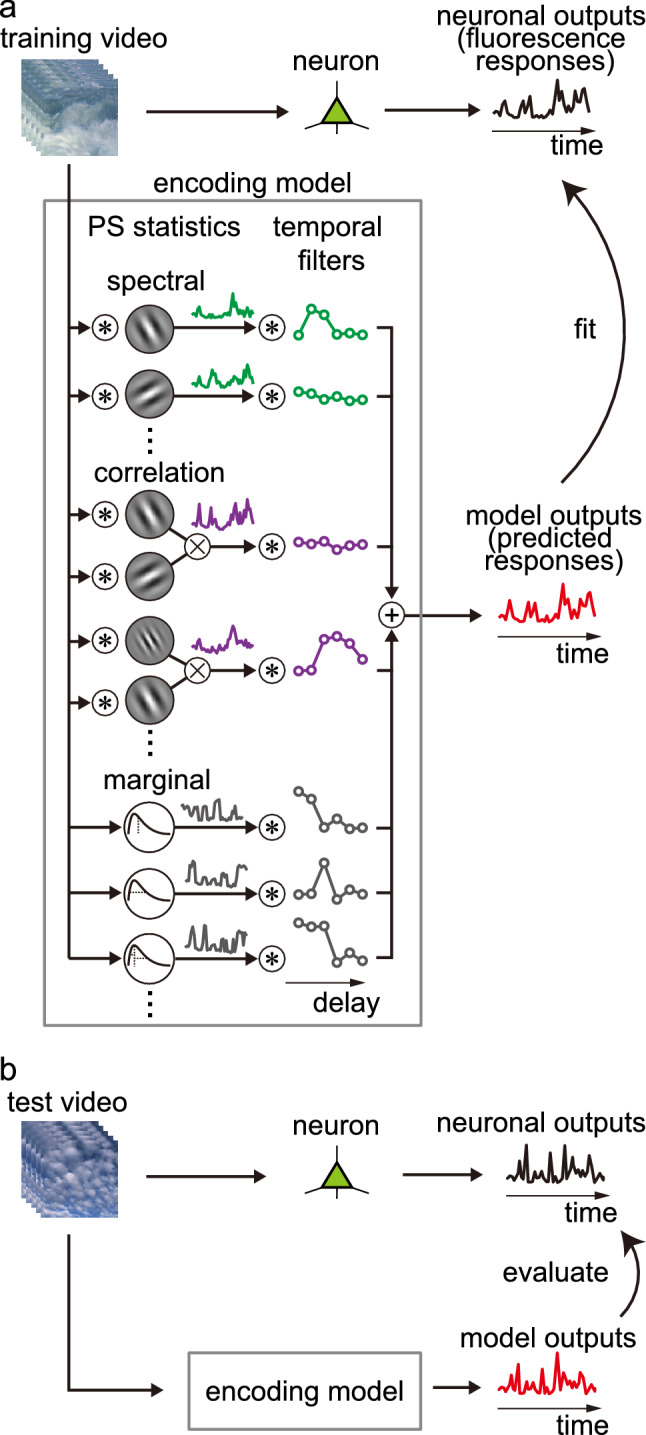
Encoding model with first-stage filters computing Portilla–Simoncelli statistics. **a** First stage of the encoding model consists of a set of spectral filters, correlations between outputs of different filters, and the marginal statistics of the luminance distributions of images (PS statistics). These filters produce time-varying outputs when a video is fed as an input. Each of these filters is followed by a temporal filter at the second stage. The model then linearly sums the second-stage filter outputs to produce the model response. For each neuron recorded, we trained the model by optimizing the temporal filters to mimic the neuron’s fluorescence responses to a training video. **b** Model performance was evaluated by comparing the model response and the neuron response to a test video that was not used for the model construction. Encircled asterisks, convolution; encircled crosses, correlation; encircled plus sign, linear summation

### In vivo calcium imaging

We performed in vivo calcium imaging of neuronal responses to naturalistic videos (see below) in V1 (17 sites) and V4 (16 sites). We recorded nine V1 sites and nine V4 sites in one animal, and eight V1 sites and seven V4 sites in the other animal. At some imaging sites, we identified the lunate sulcus, inferior occipital sulcus, and superior temporal sulcus through the observation window. We also made multiple-unit recordings to determine their minimum response fields. We further measured the distance of the imaging sites from the external meatus. Based on this set of information, we identified the areas (i.e., V1 or V4) of our recording sites. In every imaging experiment, we drilled a hole in the skull at a new position to create an imaging window. There was no overlap between the imaging sites, and hence no neurons were imaged and counted multiple times. For each animal and each hemisphere, we performed recordings first in V4, and then in V1 on later experimental days. We used this procedure rather than interleaved recordings from V1 and V4 from day to day to avoid possible deteriorating effects on V4 responses by preceding recordings in V1.

We pressure-injected a membrane-permeable fluorescent calcium indicator, Cal-520 AM (Cal-520, AAT Bioquest, Sunnyvale, CA; Tada et al. 2014), at a depth of 150–310 μm from the cortical surface through a micropipette. This depth corresponds to layer 2 and the uppermost tier of layer 3 (see Fig. 7 of Xu et al. 2003). Cal-520 was dissolved at a concentration of 1.0 mM in a solution containing 0.2% Pluronic F-127 (Thermo Fisher Scientific, Waltham, MA), 2.5% dimethyl sulfoxide (Sigma-Aldrich, St. Louis, MO), 10 mM HEPES, 2.5 mM KCl, and 150 mM NaCl (pH 7.4). We injected sulforhodamine 101 (SR 101; 100 μM, Thermo Fisher Scientific) together with Cal-520 to identify astrocytes (Nimmerjahn et al. 2004). A two-photon microscope (MOM, Sutter, Novato, CA) equipped with a mode-locked Ti:sapphire laser (MaiTai, Spectra Physics, Santa Clara, CA) was used for imaging fluorescence responses. The microscope was equipped with a 16 × objective lens (CFI75 LWD 16 × W, NA 0.8, Nikon, Tokyo), and controlled by a resonant scanner for the X direction, a galvano-scanner for the Y direction, and MOM Computer System and Software 2.0 (Sutter). The image sampling rate was 31 Hz. In V1, the imaged areas were 315 µm × 315 µm (eight sites), 355 µm × 355 µm (eight sites), or 630 µm × 630 µm (one site). In V4, the imaged areas were 315 µm × 315 µm (seven sites), 355 µm × 355 µm (seven sites), or 630 µm × 630 µm (two sites).

### Visual stimulation

We presented naturalistic videos (10° × 10°) on a gray background (36° × 20°, horizontal × vertical) to the eye contralateral to the imaged hemisphere using an organic electroluminescence display (PVM-1741, Sony, Tokyo) or a liquid crystal display (MDT231WG, Mitsubishi Electric, Tokyo). Both V1 and V4 were recorded with the same experimental setup. We used the Sony monitor for the first monkey, and the Mitsubishi monitor for the second monkey. The center portion of the videos covered the minimum response fields of the neurons that had been determined earlier by recording either multiple-unit electrical activities or fluorescence responses to presentations of 2° × 2° square patches of naturalistic videos at varying positions. The videos were composed of short clips of movies (~ 10 s) showing different scenes including landscapes, animals, and humans, as well as letters and sentences (Nishimoto and Gallant 2011; Nishimoto et al. 2011; available at https://crcns.org/data-sets/vc/vim-2/about-vim-2). Each image frame of the naturalistic movie was updated at 24 Hz. The physical refresh rate of the display was 24 Hz (Sony) or 72 Hz (Mitsubishi).

To train the encoding model, we prepared a 30-min video by concatenating the short clips to eliminate gaps, and then divided it into three 10-min videos (training sets, 10 min × three videos = 30 min). To test the quality of the model, we prepared a 3-min video that was not used for model training, and divided it into three 1-min videos. The videos were copied 10 times (1 min × three videos × 10 repetitions) and then pseudo-randomly reorganized into three 10-min videos (test sets, 10 min × three videos = 30 min). The 1-min videos appeared three or four times in each 10-min video. The training and test sets were interleaved with intervals of 1 to 10 min.

### Calcium imaging processing

In the first image-processing step, we corrected constant image distortion caused by non-linear resonant scanning by mapping the pixels to correct positions using a template of the resonant scanner position against time. We then used two-dimensional cross-correlation to adjust image displacement caused by motion of the brain (Guizar-Sicairos et al. 2008). We quantified the fluorescence from individual neurons by selecting the cell-body pixels for each neuron from the average across all acquired images and then averaging the fluorescence across the cell-body pixels. We obtained the time courses of fluorescence responses by repeating this procedure for all sampled images. The fluorescence data were high-pass filtered with a cutoff frequency of 0.02 Hz to remove slow fluctuations of signal strength that were caused by movement of the cortex in depth direction or by gradual photobleaching over recording sessions. We normalized the changes (d*F*) in fluorescence signals by the DC component (*F*_0_) of the fluorescence $(\frac{\mathrm{d}F}{{F}_{0}}=\frac{F-{F}_{0}}{{F}_{0}})$. The DC component was estimated by applying the median filter to raw signals (filter width = 50 s).

We evaluated the consistency of responses across 10 test video presentations by calculating e*xplainable variance* (Sahani and Linden 2003) as follows:

$$\overline{y}\left( t \right) = \frac{1}{N}\mathop \sum \limits_{n = 1}^{N} y_{n} \left( t \right),$$

$${\text{Explainable }}\;{\text{variance}} = 1 - \frac{{{\text{Var}}\left( {y\left( t \right) - \overline{y}\left( t \right)} \right)}}{{{\text{Var}}\left( {y\left( t \right)} \right)}}.$$

where ${y}_{n}(t)$ is the fluorescence signal (d*F*/*F*_0_) for a time bin in the *n*th trial, *N* is the number of trials, and $\mathrm{Var}(\cdot )$ denotes the variance. Explainable variance indicates the proportion of variance of fluorescence signals $y(t)$ that can be explained by stimulus-related components $\overline{y }(t)$. An explainable variance of 1 indicates the same fluorescence responses across repeated presentations of an identical video. An explainable variance of 0 indicates inconsistent fluorescence responses across trials.

### Extraction of Portilla–Simoncelli statistics from naturalistic videos

We computed PS statistics, each reflecting a different feature of a visual scene (Fig. 1a; Portilla and Simoncelli 2000), over the entire image area (10° × 10°) of each movie frame (excluding the surrounding gray region). We took this approach for two reasons. First, visual responses of neurons in V4 and even V1 are influenced by stimuli far outside the minimum response field. The range of such surrounding effects can extend beyond 20° in V1 and V4 neurons (e.g., Zhou et al. 2000). Second, the overall profile of PS statistics calculated for the entire image is affected to only a minor degree by motion in the image as long as moving objects remain inside the image.

We classified PS statistics into three groups: spectral, correlation, and marginal statistics. The spectral group of PS statistics captures the magnitudes of different orientations and spatial frequencies within an image. First, the videos were converted into the Commission International de l’Éclairage (CIE) L*a*b* color space, and only their luminance (L*) information was input into Gabor-like linear filters. Then energy filter outputs were produced by computing the amplitudes of the outputs of phase-shifted linear filter pairs. Thus, spectral statistics included only energy filter outputs averaged across an image. Correlation statistics consist of correlations of outputs of Gabor-like linear filters between different positions (linear position), orientations (linear orientation), and scales (linear scale), as well as correlations of outputs of energy filters between different positions (energy position), orientations (energy orientation), and scales (energy scale). Note that scale correlations were computed for combinations of different orientations as well as same orientations. Correlation statistics capture aspects of the image structure such as angled or curved contours, sharp edges, and periodic textural patterns involving a combination of different orientations, spatial frequencies, or positions (Portilla and Simoncelli 2000; Freeman et al. 2013). Marginal statistics refer to the summary statistics of the pixel luminance distribution of an image, such as the mean, variance, skewness, kurtosis, minimum value, and maximum value. Skewness and kurtosis were also computed for low-pass images at different cutoff frequencies (approximately ~ 18, ~ 9, ~ 4.5, and ~ 2.3 cycles/°; in order from higher to lower: Skew. L1, Skew. L2, Skew. L3, and Skew. L4; Kurt. L1, Kurt. L2, Kurt. L3, and Kurt. L4). For high-pass images (18 cycles/° ~), the variance was computed (Var. HP).

We computed spectral and correlation statistics using six orientations (0°, 30°, 60°, 90°, 120°, and 150°), three spatial frequency bands (center frequencies, 12.8, 6.4, and 3.2 cycles/°; band width, approximately one octave), and 7 × 7 position shifts. Spectral statistics of high-pass (18 cycles/° ~) and low-pass (~ 2.3 cycles/°) images were also included without decomposing into orientation bands. A total of 891 PS statistics were computed for a single video frame (spectral, 20; marginal, 15; linear position, 100; linear orientation, 45; linear scale, 144; energy position, 450; energy orientation, 45; energy scale, 72; see Table 1 for derivation of the number of each type of PS statistic). We obtained the time courses of the PS statistics by concatenating them across video frames, and then compressed them to 6 Hz by averaging every four consecutive frames. Individual statistics were normalized by the mean and standard deviation, i.e., *z*-scored. To compute PS statistics, we used the code provided on the website of the Simoncelli laboratory (http://www.cns.nyu.edu/~lcv/texture/) with some modifications (i.e., removing linear scale and orientation correlations for non-oriented, low-frequency features).

**Table 1:** Components of Portilla–Simoncelli statistics

Group		Count	Note
Spectral	S	20	6 orientations × 3 scales + 1 high pass + 1 low pass
Marginal	M	15	mean, variance, skewness, kurtosis, min, max, variance (1 high pass), skewness (4 low pass), kurtosis (4 low pass)
Linear position	LP	100	Combination of 7 × 7 position shifts (25) × 4 scales
Linear orientation	LO	45	Combination of 6 orientations (15) × 3 scales
Linear scale	LS	144	6 orientations × 12 orientations (odd and even filters) × 2 scale pairs
Energy position	EP	450	Combination of 7 × 7 position shifts (25) × 6 orientations × 3 scales
Energy orientation	EO	45	Combination of 6 orientations (15) × 3 scales
Energy scale	ES	72	6 orientations × 6 orientations × 2 scale pairs
Total		891	

Combination of 7 × 7 position shifts (25): $\frac{7 \times 7 - 1}{2} + 1 = 25$

Combination of six orientations (15): $_{6} C_{2} = 15$

To evaluate how temporal factors of the stimuli influence the results, we computed flicker/motion energy within a small region of the display across the movies to test its covariation with the PS statistics across the entire area. We used a bank of spatiotemporal Gabor filters (see Nishimoto et al. 2011). Filters were tiled to cover the entire area of the video images (10° × 10°) with spacing depending on the spatial Gaussian envelope. The full set contains 6,555 Gabor filters. Among them, 1,425 filters had no temporal component (temporal frequency = 0) and were excluded from the following analysis. For calculating flicker/motion energy, we used the remaining 5,130 filters including those with motion component (temporal frequency > 0) with five spatial frequencies (0.2, 0.4, 0.8, 1.6, 3.2 cycles/°), two temporal frequencies (3.2, 6.4 Hz), and eight directions (45° apart) as well as those with flickering component (temporal frequency > 0, spatial frequency = 0). Flicker/motion energy for each parameter set (i.e., position, orientation, and spatial frequency) was obtained by convolving the video frames with three-dimensional (spatiotemporal) Gabor filters across 10 frames. We then calculated Pearson’s correlation coefficients between flicker/motion energy for each parameter set and the PS statistics (calculated for the entire image) over the duration of videos (33 min: 30 min training video and 3 min test video). We divided the PS statistics into the spectral group (*n* = 20) and non-spectral group (*n* = 871). We also examined the correlation between the PS statistics and the sum of flicker/motion energy (calculated from Gabor filters with the same direction but with different spatial frequencies, temporal frequencies, and positions) to see if a global sum of motion energy across the image correlated with the PS statistics.

### Encoding-model analysis

We characterized the response selectivity of each neuron to a variety of PS statistics by fitting an encoding model to the fluorescence signals evoked by the naturalistic videos (Fig. 1). An array of linear temporal filters received outputs of the corresponding PS statistics filters (Fig. 1a). These temporal filters, spanning delays from 0 to 1 s at an interval of 1/6 s, were used to reproduce the delayed contributions of the PS statistics time courses to the fluorescence signals (Smetters et al. 1999; Ikezoe et al. 2018). The signals were then linearly summed across the filters to produce the model outputs.

We modeled fluorescence signals for individual neurons by optimizing temporal filters (Fig. 1a) at the second stage using L2-regularized linear regression (ridge regression; Huth et al. 2012). Fluorescence signals were normalized by the mean and standard deviation in each neuron to compensate for differences in signal strength across neurons. To optimize regularization parameters, we divided the entire 30-min training data into 50 chunks (each chunk = 36 s), and randomly picked 90% of the chunks for regression. For each recording site, we chose the regularization parameter that achieved the highest response-prediction accuracy for the remaining 10% of the data (the optimal regularization parameter). The final model was then obtained by regressions that included all training data and the optimal regularization parameter. The performance of the constructed model was tested using another video (test video; 3 min) that had not been used for model construction (Fig. 1b). Neuronwise modeling accuracy (prediction performance) was quantified using Pearson’s correlation coefficient (*r*) between the measured and predicted fluorescence signals for the test set.

To quantify the relative contribution of marginal and correlation statistics over spectral statistics, we computed the non-spectral weight ratio by dividing the mean absolute value of the second-stage filter weights of correlation statistics and marginal statistics by the mean absolute value of the weights of all PS statistics.

We applied receiver operating characteristic (ROC) analysis to quantify the differences between V1 and V4 (Green and Swets 1966). We computed the area under the curve (AUC) that represented the degree of separation between two distributions. An AUC of 0.5 indicates that the weight distributions of V1 and V4 are totally overlapped and indistinguishable. An AUC smaller than 0.5 indicates that V4 weights are smaller than V1 weights, on average. An AUC of 0.0 indicates that two distributions are perfectly separated, and the maximum V4 weight is smaller than minimum V1 weight. If an AUC is larger than 0.5, V4 weights are larger than V1 weights, on average. Finally, an AUC of 1.0 means that two distributions are perfectly separated but the order is reversed, i.e., the minimum V4 weight is larger than the maximum V1 weight.

## Results

### Reliability of fluorescence responses of individual neurons to naturalistic videos

**Fig. 2:**
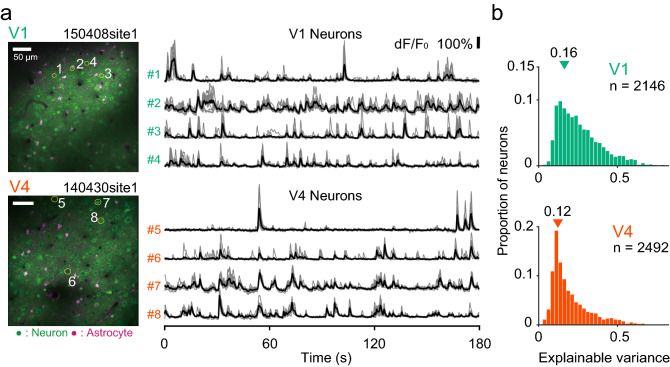
Reliability and diversity of fluorescence responses of V1 and V4 neurons to naturalistic videos. **a** Fluorescence response time courses of example neurons in V1 (#1 to #4) and V4 (#5 to #8). Black lines indicate average responses across 10 trials, and gray lines indicate responses in individual trials. Numbers in the two-photon microscopy images correspond to the neuron numbers to the left of the fluorescence traces. In contrast with astroglia, neurons were sulforhodamine negative. d*F*/*F*_0_ denotes changes (d*F*) in fluorescence signals normalized by the DC component of the fluorescence (*F*_0_). **b** Frequency histograms of the explainable variance of our neuronal samples from V1 (top) and V4 (bottom). Triangles represent the median value of each area (V1, 0.16; V4, 0.12)

We recorded fluorescence responses to naturalistic videos from 2146 neurons in V1 (17 sites) and 2492 neurons in V4 (16 sites) of two monkeys. Fluorescence strength dynamically changed during the presentation of the videos. Figure 2a shows fluorescence responses of example neurons in V1 (#1 to #4) and V4 (#5 to #8). Gray traces represent responses of individual trials and black traces represent the trial average across 10 stimulus presentations. Each neuron consistently responded to the videos at roughly the same time point across trials. To determine how well the fluorescence signals reflected neuronal stimulus selectivities rather than response fluctuations caused by internal and recording noise, we quantified the consistency of the fluorescence signals across trials by computing the explainable variance (Sahani and Linden 2003; see “Materials and methods”). It ranged from 0.029 to 0.77 in V1 and from 0.013 to 0.71 in V4 (Fig. 2b; medians, 0.16 for V1 and 0.12 for V4), meaning that 16% and 12% of the variance in the responses in V1 and V4, respectively, could be explained by the variety of video images, and thus reflected neuronal visual selectivities. The explainable variance was larger in V1 than V4 (Wilcoxon rank-sum test, *p* < 10^−5^, AUC = 0.33), indicating that the signals were more robust against noise in V1 than in V4.

Unexplainable noise contains both neural and non-neuronal noise. We evaluated fluorescence fluctuation during the first 5 s of each trial when no visual stimulus was shown. Although the fluorescence fluctuation during this period included the contribution of spontaneous activity, we took this variation as a proxy for non-neuronal noise. We then calculated the signal-to-noise ratio by dividing the maximum fluorescence response by this fluorescence fluctuation. The signal-to-noise ratio was slightly higher in V1 than in V4 (V1, median 15.9, interquartile range, 16.3; V4, 14.2 and 14.3, respectively; Wilcoxon rank-sum test, *p* < 10^−5^, AUC = 0.44). The results suggest that the difference in explainable variance between V1 and V4 only partially reflects a difference in non-neuronal noise, and that to a greater degree it indicates trial-by-trial variability in neural responses.

### Prediction performances of PS models

**Fig. 3:**
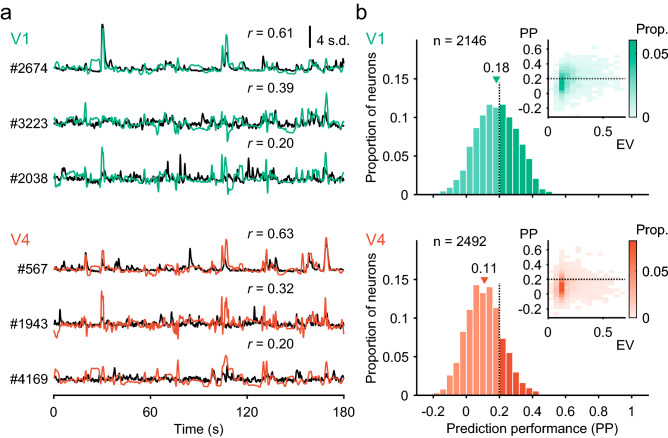
Prediction performance of the encoding models with Portilla–Simoncelli statistics filters. **a** Average responses of V1 and V4 neurons (black traces) to a 3-min test video across 10 trials and the predicted responses (green for V1, orange for V4). The prediction performance (*r*) is shown for each neuron. The scale indicates the response strength equivalent to 4 standard deviations (SD) of the *z*-scored signal strength distribution. **b** Frequency histograms of prediction performance for our entire database (*n* = 2146 neurons for V1, *n* = 2492 neurons for V4). Triangles represent the median values. Neurons exceeding > 0.2 performance accuracy (dark columns) were further analyzed. Insets show the scatter plots of prediction performance against explainable variance. Prediction performance was positively correlated with explainable variance in V1 and V4

For each neuron, we constructed an encoding model based on PS statistics to characterize the neuronal responses to naturalistic videos. We sought to simulate responses to a training video of 30 min by defining the optimal set of weights for PS statistics (Fig. 1a). The performance of the constructed model was tested using another video (test video; 3 min) that had been kept unused for model construction (Fig. 1b). The model response to the test video varied along the time axis as the contents of the video changed (Fig. 3a; V1, green traces; V4, orange traces). The time course of the model output depended on the visual selectivity of the corresponding neurons, and thus differed across the models. For the successful example neurons shown in Fig. 3a (#2674 in V1, #567 in V4), the outputs of the models faithfully followed the fluorescence responses of the corresponding neurons (Fig. 3a, black traces). Note that neuronal responses were averaged across 10 trial repetitions to minimize the effects of noise.

The prediction performances of the models as evaluated by Pearson’s correlation coefficients between the model outputs and the neuronal responses were 0.61 for the V1 neuron and 0.63 for the V4 neuron. In less successful examples (#3223 in V1, #1943 in V4), the model outputs moderately followed the neuronal responses and the prediction performances for V1 and V4 were 0.39 (#3223) and 0.32 (#1943), respectively. The lowest traces for V1 (#2038) and V4 (#4169) represent example neurons with a correlation coefficient of 0.20, which we defined as the inclusion criterion for further analyses (see below). The mean prediction performances across neurons were 0.18 ± 0.13 (*n* = 2146, mean ± s.d.) in V1 and 0.11 ± 0.11 (*n* = 2492) in V4. The above results were based on the calculation of PS statistics across the entire stimulus image (10° × 10°). We performed the same analysis for a central image area of 5° × 5°. Note that this central area contained the minimum response field determined for multiple-unit activity at each imaging site. Prediction performance based on 5° × 5° was highly correlated with that based on 10° × 10° (V1, *r* = 0.86, *p* < 10^−5^; V4, *r* = 0.76, *p* < 10^−5^). In V1, prediction performance was increased to 0.21 ± 0.13 (*n* = 2146, *t* test, *p* < 10^−5^, *d* = 0.15). In V4, prediction performance did not depend on the image area analyzed (0.11 ± 0.11, *n* = 2492, *p* = 0.18, *d* = 0.027).

The distributions of the prediction performance were broad in both areas (Fig. 3b), indicating that some models predicted the responses relatively accurately but other models did so only moderately or poorly. The prediction performance was positively correlated with the explainable variance (insets in Fig. 3b; Spearman’s correlation coefficient *r*_*s*_ = 0.39 in V1, *r*_*s*_ = 0.17 in V4, *p* < 10^−5^), indicating that as expected, the models worked better for neurons with more robust responses across trials. Despite the variety across the models, on a population-wide level the prediction performance was significantly higher than zero both in V1 and V4 (*t* test, *p* < 10^−5^). Our encoding-model approach captured the visual selectivity of neuronal subpopulations by means of PS statistics. The prediction performance was higher in V1 than V4 (*t* test, *p* < 10^−5^, *d* =  − 0.611). For the following analyses, with the exception of Fig. 8b, c, we chose neurons with prediction performances exceeding 0.2 (dark columns in Fig. 3b; 963 neurons in V1, 488 neurons in V4). Changing this selection criterion from 0.2 to 0.1 or 0.3 did not affect the overall conclusions of this study. The sizes of minimum response fields of these neurons, estimated as the standard deviation of two-dimensional Gaussian fitting to the response map in *in-silico* simulations (Nishimoto et al. 2011; Ikezoe et al. 2018), were 1.13° ± 0.80° (mean ± SD) in V1 and 2.28° ± 1.91° in V4. The receptive fields were twice as large in V4 as in V1 (*p* < 10^−5^; Wilcoxon rank-sum test).

### Comparison of tunings for image statistics in V1 and V4

We examined how neuronal tunings to PS statistics contributed to responses to naturalistic videos. In this analysis, we grouped the PS statistics into eight categories: spectral statistics, marginal statistics, and correlations between linear positions, linear orientations, linear scales, energy positions, energy orientations, and energy scales (Table 1). Each category contained a number of statistics, and each statistic had its own fitting weight. To evaluate the contributions of the image statistics, we used the absolute value of the second-stage filter weights. The second-stage filters were temporal filters with six weight values across time. We calculated the mean of the absolute values of temporal filters of each PS statistic, then chose the largest value for each category to represent the importance in the neuronal responses.

**Fig. 4:**
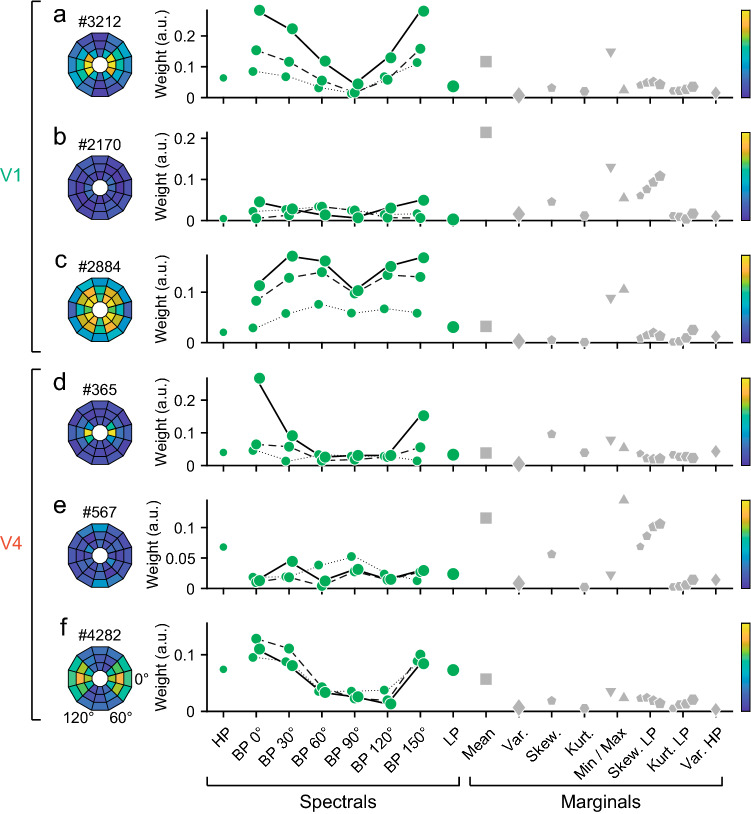
Contributions of different PS statistics groups to single neuron responses. Fitting weights are plotted for the parameters of 20 spectral statistics (high pass + six orientations × three band passes + low pass) and 15 marginal statistics (mean, variance, skewness, kurtosis, minimum, maximum, skewness × four low passes, kurtosis × four low passes, variance for high pass) in representative neurons in V1 (**a–c**) and V4 (**d–f**). Spectral statistics consist of a high-pass filter (HP), oriented bandpass filters (BP 0° to BP 150°), and a low-pass filter (LP). Oriented bandpass filters have three spatial scales, the data of which are connected with lines across orientations (center frequencies; 3.2, 6.4, and 12.8 cycles/° for solid, dashed, and dotted lines, respectively). Marginal statistics consist of mean, variance (Var.), skewness (Skew.), kurtosis (Kurt.), minimum (Min) and maximum (Max), and skewness and kurtosis for four low-pass filtered images (Skew. LP, Kurt. LP), and variance for a high-pass filtered image (Var. HP). Colored circles on the left show weight distributions for orientation and spatial frequency in the Fourier domain; the angle indicates orientation and the radius indicates spatial frequency (from low frequency in the center towards high frequency in the outer). Color bars on the right indicate scales for weight that correspond to the vertical axis of graphs on the same rows of the center panel

**Fig. 5:**
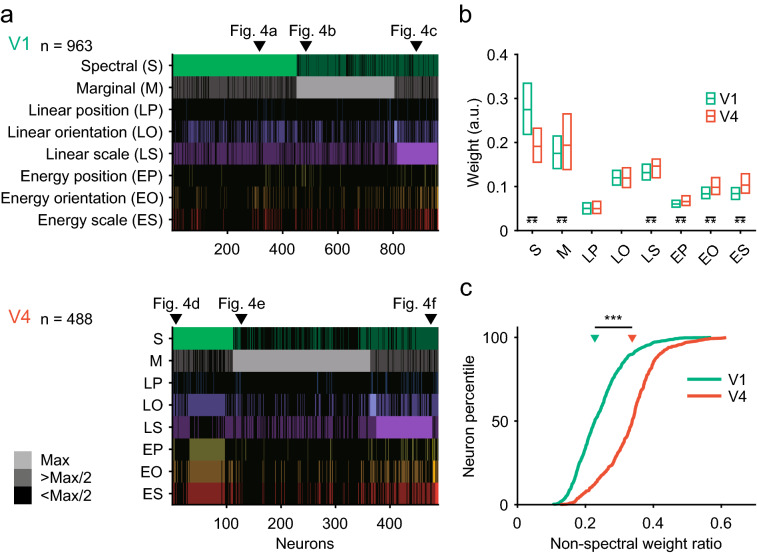
Comparison of fitting weights between V1 and V4. **a** Fitting weights of eight groups of image statistics are plotted for each neuron examined in V1 and V4 (*n* = 963 for V1, *n* = 488 for V4). Different colors represent different image statistics groups. The brightness represents the weight amplitude; for each neuron, the greatest brightness indicates maximum weight (Max), the intermediate brightness indicates intermediate weight (more than half-maximum: > Max/2), and the black areas indicate low weight (less than half-maximum: < Max/2). The neurons shown in Fig. 4 are indicated by triangles with the figure numbers. **b** Box plots of fitting weights of the eight statistics groups. The horizontal line in each box indicates the median, the upper edge indicates the 75th percentile, and the lower edge indicates the 25th percentile. Asterisks indicate a statistically significant difference (**p < 0.01/8; Wilcoxon rank-sum test, Bonferroni correction). a.u.: arbitrary unit. **c** Cumulative distributions of the non-spectral weight ratio (see “Materials and methods”) for V1 and V4. Triangles indicate the median values (V1, 0.24; V4, 0.34). Asterisks (***) indicate a significant difference between the two areas (*p* < 10^−5^; Wilcoxon rank-sum test). *S* spectral, *M* marginal, *LP* linear position correlation, *LO* linear orientation correlation, *LS* linear scale correlation, *EP* energy position correlation, *EO* energy orientation correlation, *ES* energy scale correlation

Figure 4 shows responses of prototypical single neurons. Figure 5 summarizes the population data for V1 and V4. In many neurons in V1 (47%, 449/963 neurons), spectral statistics had the largest fitting weight (Figs. 4a, 5a, upper panel), as we would expect given the well-known tunings of V1 neurons to spatial frequency and orientation. The example neuron in Fig. 4a had large weights modulated by both orientation and spatial frequency, but the contributions of marginal statistics were relatively small. In another large population of V1 neurons (37%, 355/963), responses depended critically on marginal statistics. An example neuron from this group (Fig. 4b) was insensitive to orientation and spatial frequency, but was modulated by the mean, minimum, maximum, and skewness of low-pass features of the luminance distribution. In a small subset of V1 neurons (15%, 144/963), linear scale correlation had the largest weight. An example neuron from this group (Fig. 4c) was sensitive to spatial frequency and the minimum and maximum of the luminance distribution, but was not sensitive to orientation or the other marginal statistics. The responses of 1.0% of V1 neurons (ten neurons) were best explained by linear orientation correlation. All other groups of statistics, such as linear position correlation and the three energy correlations, contributed little to the responses of V1 neurons.

In contrast, more than half of V4 neurons were influenced by marginal statistics (Fig. 4e; 52%, 252/488). The proportion of neurons in which spectral statistics had the largest weight (23%, 111/488) was relatively small compared to V1 (Fig. 5a, lower panel). Figure 4d shows an example V4 neuron with sensitivity to orientation. In addition, unlike V1 neurons, many of the V4 neurons that were tuned to spectral statistics were also influenced by higher order statistics, including energy correlations; the weights of these correlations were larger than the half-maximum weight (see bright colors in the rows for energy position, energy orientation, and energy scale in Fig. 5a, lower panel). In a smaller population of V4 neurons (21%, 103/488) (e.g., Fig. 4f), linear scale correlation had the largest weight; the percentage of these neurons was higher than that in V1 (15%). A minority of V4 neurons were preferentially tuned to other linear or energy correlations (4.5%, 22/488). We obtained similar results when examining the peak (maximum), instead of the mean, of the temporal filter weights for characterizing the contribution of each PS statistic category.

The overall fitting weight profiles across the eight categories of PS statistics were similar between V1 and V4 (Fig. 5b). The spectral and marginal statistics had the largest weights, followed by linear scale correlation. More detailed comparisons between V1 and V4 revealed that the weight of spectral statistics was larger, on average, in V1 than in V4 (Wilcoxon rank-sum test, Bonferroni correction for multiple comparisons across the eight groups; *p* < 10^−5^/8, AUC = 0.22). In contrast, the weight of marginal statistics was larger in V4 than in V1 (*p* = 6.6 × 10^−5^, AUC = 0.56, Fig. 5b). The weights of correlation statistics were generally larger in V4 than in V1. Specifically, the weights of the linear scale correlation (*p* < 10^−5^/8, AUC = 0.59), energy position correlation (*p* < 10^−5^/8, AUC = 0.62), energy orientation correlation (*p* < 10^−5^/8, AUC = 0.69), and energy scale correlation (*p* < 10^−5^/8, AUC = 0.72) were slightly larger in V4 than in V1. The weight of the linear scale correlation was the largest among the correlation statistics in V1 and V4.

We computed non-spectral weight ratios (i.e., the mean absolute value of the second-stage filter weights of marginal statistics and correlation statistics divided by the sum of the mean absolute value of the weights of spectral statistics and that of marginal and correlation statistics) to quantify how marginal and correlation statistics compared to spectral statistics in terms of their contribution to neuronal responses. The ratios were higher in V4 than in V1 (Fig. 5c; V4, median = 0.34, interquartile range = 0.096, *n* = 488; V1, 0.23 and 0.097, respectively, *n* = 963; Wilcoxon rank-sum test, *p* < 10^−5^, AUC = 0.80). A possible complication of the comparison between V1 and V4 was the difference in explainable variance (Fig. 3b) between the two areas. To address this concern, we conducted an analysis in which we selected neurons with a matched range of explainable variance (0.2–0.5), and compared the non-spectral weight ratio between V1 and V4. As shown for all the data in the original analysis (Fig. 5c), the non-spectral weight ratio was higher in V4 than in V1 for these samples (V4, median = 0.31, interquartile range = 0.093, *n* = 113; V1, 0.23 and 0.097, respectively, *n* = 401; Wilcoxon rank-sum test, *p* < 10^−5^). These findings demonstrate that tunings to marginal and correlation statistics were more explicit in V4 than in V1.

**Fig. 6:**
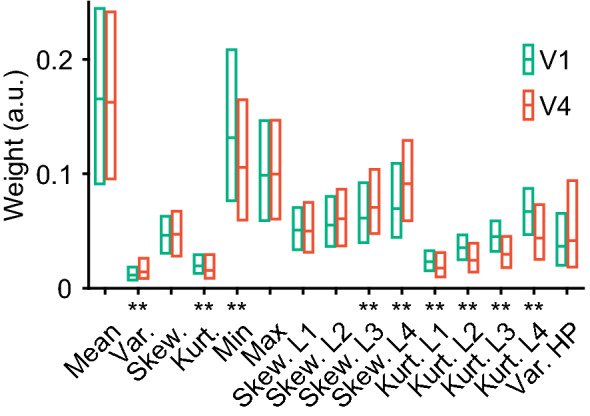
Fitting weights of different parameters of marginal statistics in V1 and V4. Fitting weights are plotted for various parameters of the luminance distribution (marginal statistics), including the mean, variance (Var), skewness (Skew), Kurtosis (Kurt), Minimum (Min), Maximum (Max), sub-band Skewness (Skew. L1 to Skew. L4), sub-band Kurtosis (Kurt. L1 to Kurt. L4), and variance of high-pass filtered images (Var. HP). See “Materials and methods” for details. Asterisks (**) indicate a significant difference between the two areas (*p* = 0.01/15; Wilcoxon rank-sum test with Bonferroni correction for multiple comparisons for the 15 marginal statistics). a.u., arbitrary unit

Because marginal statistics contain a variety of parameters (i.e., mean, variance, skewness, kurtosis, and range), we next analyzed the weight strength of each parameter, as shown in Fig. 6. The weights in this figure are absolute values, hence they are > 0. To evaluate the statistical relevance of these values, we compared these weights with the minimum weight among all PS statistics. The assumption here was that the minimum weight was at or higher than noise level. We found that the weights of all marginal statistics were greater than the minimum weight of all PS statistics (Wilcoxon’s rank-sum test with Bonferroni correction for multiple comparisons across 15 marginal statistics, *p* < 0.001/15), suggesting that they were statistically significant.

The weight of the mean of the luminance histogram (Mean) was comparable between V1 and V4 (Wilcoxon rank-sum test, Bonferroni correction across 15 marginal statistics; *p* > 0.05/15, AUC = 0.50), and larger than those of the other parameters in each area. The weights of minimum and kurtosis values computed from the original (Kurt, *p* < 10^−5^/15, AUC = 0.43) and low-passed images (Kurt. L1, *p* < 10^−5^/15, AUC = 0.40; Kurt. L2, *p* < 10^−5^/15, AUC = 0.35; Kurt. L3, *p* < 10^−5^/15, AUC = 0.31; Kurt. L4, *p* < 10^−5^/15, AUC = 0.35) were larger in V1 than in V4, whereas the weights of the variance (Var, *p* < 10^−5^/15, AUC = 0.60) and some skewness values computed from coarser low-passed images (Skew. L3, *p* = 3.1 × 10^−5^, AUC = 0.57; Skew. L4, *p* < 10^−5^/15, AUC = 0.61) were larger in V4 than in V1. No differences were found between V1 and V4 regarding skewness from the original (Skew, *p* > 0.05/15, AUC = 0.51), maximum (Max, *p* > 0.05/15, AUC = 0.50), skewness computed from finer low-passed images (Skew. L1, *p* > 0.05/15, AUC = 0.50; Skew. L2, *p* > 0.05/15, AUC = 0.52), or variance of high-pass filtered images (Var. HP, *p* > 0.05/15, AUC = 0.54).

Among the 15 parameters of marginal statistics, the weights were dominated by mean, maximum, and minimum, both in V1 and V4 (Fig. 6). One may wonder whether these strong weights were found in the same neurons or different neurons. Scatter plot analysis indicated no or weak negative correlation between the absolute weight for either maximum or minimum luminance and the absolute weight for mean luminance (Max vs. Mean: *r*_*s*_ =  − 0.15, *p* < 0.01 for V1, *r*_*s*_ =  − 0.14,* p* < 0.01 for V4; Min vs. Mean: *r*_*s*_ = 0.06, *p* = 0.08 for V1, *r*_*s*_ =  − 0.17 for V4, *p* < 0.01). These findings indicate that the strong weights for the mean, maximum, and minimum of the luminance did not arise in the same neurons; they arose either randomly across neurons or separately in different neurons.

The PS statistics include only spatial information embedded in the naturalistic videos and do not capture temporal factors, such as local motion energy. If the PS statistics and motion energy were strongly correlated, preference for the PS statistics might actually reflect that for motion energy. To address this issue, we examined whether and to what degree the PS statistics (calculated for the entire image) co-varied with flicker/motion energy by computing the correlation of temporal fluctuation of these metrics. A bank of spatiotemporal Gabor filters (*n* = 5130) was used to calculate local motion energy in the videos. The distributions of correlation coefficients were deviated towards positive values from zero in both the spectral and non-spectral groups (Wilcoxon rank-sum test, *p* < 10^−5^). The median was 0.24 (interquartile range: 0.13) for the spectral group and was 0.094 (interquartile range: 0.11) for the non-spectral group. The correlation was weaker in the non-spectral group than in the spectral group (Wilcoxon rank-sum test, *p* < 10^−5^). Similar results were obtained when we examined correlation between the PS statistics and the sum of motion energy with the same direction; the median *r* was 0.39 (interquartile range: 0.15) for the spectral group and 0.16 (0.095) for the non-spectral group. Both were deviated from zero, and the former was larger than the latter (Wilcoxon rank-sum test, *p* < 10^−5^).

### Spatial distribution of neurons with different tuning preferences

**Fig. 7:**
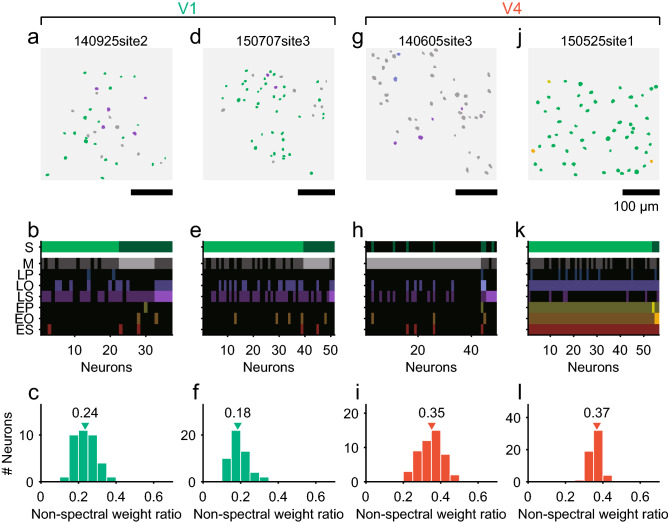
Spatial distributions of neurons with different tunings to Portilla–Simoncelli statistics in V1 and V4. **a**, **d**, **g**, **j** Spatial maps of neurons with largest weights of different Portilla–Simoncelli statistics groups in local regions of V1 (**a**, **d**) and V4 (**g**, **j**). Neurons are color-labeled according to the statistics group to which they are most sensitive. See panel **b** for the color codes. Scales indicate 100 µm. **b**, **e**, **h**, **k** Fitting weights of eight groups of image statistics are plotted for neurons imaged at sites **a**, **d**, **g**, and **j**, respectively. See Fig. 4 legend for the color codes. **c**, **f**, **i**, **l** Frequency histograms of the non-spectral weight ratio for sites **a**, **d**, **g**, and **j**, respectively. Triangles indicate the median values for individual sites. See Fig. 4a for abbreviations of statistics categories

We next examined the spatial distribution of the neurons across the cortical surfaces of V1 and V4 according to their tunings to PS statistics. By labeling each neuron according to the category with the largest fitting weight, we created a map showing which PS statistic groups contributed most in individual neurons. In an example V1 site, nearly half of the neurons (59%, 22/37) were tuned to spectral statistics, while another 27% (10/37) were tuned to marginal statistics (Fig. 7a, b). The two groups of neurons were intermingled with each other. In another V1 site, the vast majority of neurons (76%, 39/51) were tuned to spectral statistics and only a small population (20%, 10/51) were tuned to marginal statistics (Fig. 7d, e). Although 15% of V1 neurons were tuned to linear scale correlation (Fig. 5a, upper panel), we found no sites in which these neurons were predominant.

The distribution of neurons with different tuning preferences varied across the recording sites more drastically in V4 than in V1. In an example V4 site (Fig. 7g, h), most neurons (88%, 43/49) were tuned to marginal statistics and the rest were tuned to linear scale correlation. Few neurons in this site were sensitive to spectral statistics. In contrast, in another V4 recording site, most neurons (95%, 53/56) were preferentially tuned to spectral statistics (Fig. 7j, k). Note that these neurons were also sensitive to correlation statistics, and the weights of several subgroups of correlation statistics reached half the value of the largest weight of spectral statistics (Fig. 7k). The non-spectral weight ratios in V4 sites were relatively high (median of V4, 0.34; Fig. 7i, 0.35; Fig. 7l, 0.37), indicating that the responses of the neurons at these sites were influenced by marginal and correlation statistics in addition to spectral statistics. In contrast, the non-spectral weight ratios were relatively low in the V1 sites (median of V1, 0.23; Fig. 7c, 0.24; Fig. 7f, 0.18), confirming that simple features defined by spectral statistics contributed more to neuronal responses in V1 than in V4.

**Fig. 8:**
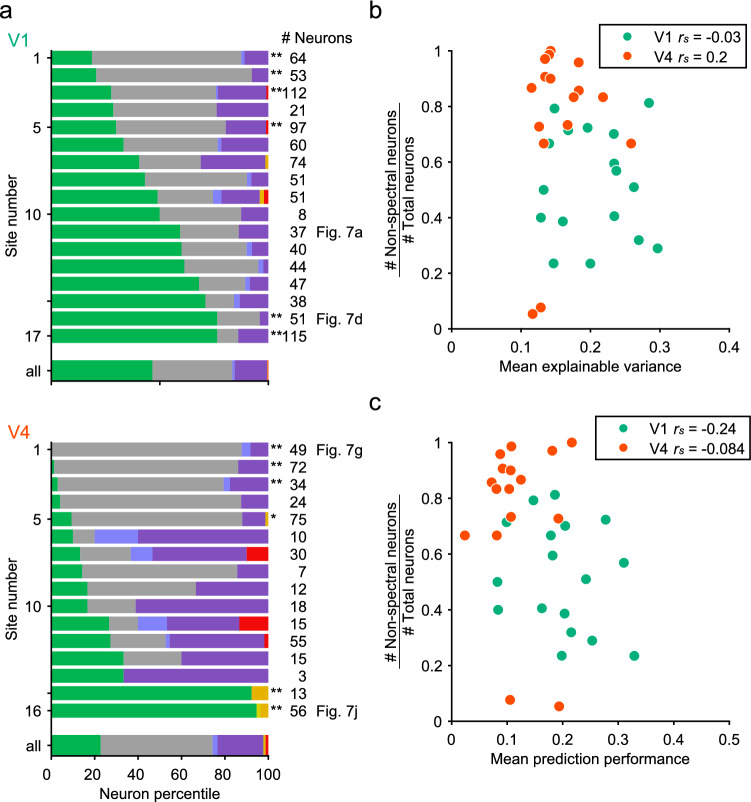
Variation of distribution of neurons tuned to spectral, marginal, and correlational statistics across imaging sites. **a** Percentages of neurons with the largest fitting weights for spectral, marginal, and correlation statistics are shown for individual sites in V1 (upper panel, *n* = 17) and V4 (lower panel, *n* = 16). The lowest row in each panel is the sum of the fractions across all imaging sites in V1 and V4, respectively. Asterisks indicate a significant deviation of neuron type distribution from the null distribution (see text for details; *p < 0.05, **p < 0.01; Bonferroni correction for multiple comparisons across the 17 sites in V1 and the 16 sites in V4). The color code is the same as in Fig. 4a. **b, c** Ratio of the number of non-spectral neurons to the total number of neurons is plotted against the mean explainable variance (**b**) and the mean prediction performance (**c**) for 17 V1 sites and 16 V4 sites. Each data point indicates data for each imaging site

The variability of the tuned PS statistics categories across imaging sites is evident in a summary figure illustrating the percentages of neurons tuned to spectral, marginal, and correlation statistics in all imaging sites (Fig. 8a). In V1, neurons tuned to spectral statistics (green bars) and those tuned to marginal statistics (grey bars) were dominant, with varying relative proportions across sites. Neurons tuned to correlation statistics (bars in blue and other colors) were a minor population in all V1 sites. In V4, neurons tuned to correlation statistics constituted more than 50% of the imaged neurons in seven sites, and less than 30% in the remaining nine sites. In two sites in V4, neurons tuned to spectral statistics were predominant (the lowest two rows in Fig. 8a). To determine whether this scattering of neuron types was random or significant, we generated null distributions of neuron types by randomly drawing neurons from all the pooled data 100,000 times and assigning them to each site, retaining the same number of neurons per site. The results of this analysis showed that the distribution of neuron types at six sites in V1 and six sites in V4 were significantly different from the null distribution (asterisks in Fig. 8a; *p < 0.05, **p < 0.01; Bonferroni correction for multiple comparisons across the 17 sites in V1 and the 16 sites in V4). These results suggest that neurons with selectivities for different PS statistics groups were not uniformly distributed, but they were locally clustered in both V1 and V4. In particular, V4 consisted of sites dominated by neurons tuned to correlation statistics and sites with only a small proportion of such neurons.

To determine if the mean explainable variance and prediction performance affected the estimation of tuning types at each recording site, we plotted the non-spectral preferred neuron ratio (the number of neurons preferring non-spectral statistics divided by the total number of neurons at each site) against the mean explainable variance (Fig. 8b) and the mean prediction performance (Fig. 8c). In both V1 and V4, the non-spectral preferred neuron ratio was not correlated to the explainable variance or prediction performance. Spearman’s correlation coefficient was − 0.03 (*p* = 0.91) in V1 and 0.20 (*p* = 0.47) in V4 for explainable variance, and − 0.24 (*p* = 0.36) in V1 and − 0.084 (*p* = 0.76) in V4 for prediction performance. Therefore, the variability of explainable variance and prediction performance is unlikely to account for the variation in neuron types across the imaging sites.

**Fig. 9:**
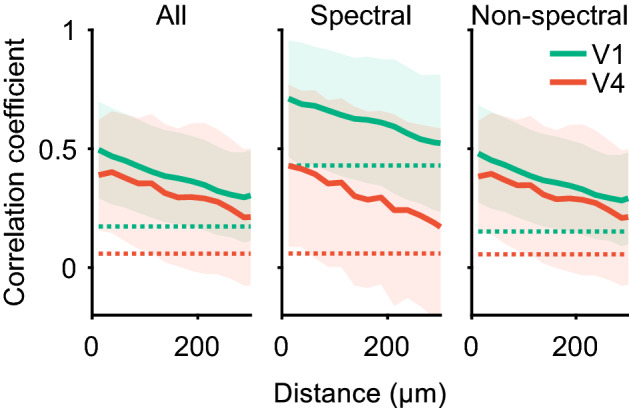
Dependence of response similarities on intercellular distance. For all pairs of neurons in V1 and V4 recording sites, Spearman’s correlation coefficients of fitting weights in three sets (all, spectral, and non-spectral) of PS statistics are plotted against the interneuronal distances. Solid lines and shaded areas indicate the mean and the standard deviation of weight correlations in each bin (25 µm). Dotted lines indicate the average of the 100,000 permutations of the shuffled data

Clustering of neurons with similar selectivities to PS statistics was also supported by the relation between intercellular distance and fitting weight similarities. We plotted Spearman’s correlation coefficients of fitting weights of all PS statistics against the distance between two neurons within single sites (Fig. 9, left panel). As the distance between a neuron pair increased, the pairwise correlation of fitting weights decreased both in V1 and V4. Spearman’s correlations (*r*_*s*_) between the pairwise correlation of fitting weight and intercellular distance were − 0.23 for V1 and − 0.23 for V4. They were confirmed to be significant by comparing them to correlations calculated for shuffled predictors (*p* < 10^−5^; test with 100,000 permutations). Similar negative correlations between the fitting weight and cellular distance were found when separately examining spectral statistics and non-spectral statistics (Fig. 9, middle and right panels; for spectral statistics, *r*_*s*_ =  − 0.17 in V1 and − 0.17 in V4; for non-spectral statistics, *r*_*s*_ =  − 0.23 in V1 and − 0.22 in V4; *p* < 10^−5^). In addition to the analysis for all sites combined, we performed a similar analysis for each site. Thirteen of 17 sites in V1 and nine of 16 sites in V4 showed a significant correlation (*p* < 0.05; Bonferroni correction for multiple comparisons across the 17 sites in V1 and the 16 sites in V4; test with 100,000 permutations).

## Discussion

By combining two-photon calcium imaging and encoding-model analysis, we characterized the response selectivities of neurons in V1 and V4 to PS statistics based on their responses to naturalistic videos. The responses of many neurons in both areas were explained most by the spectral statistics, marginal statistics, or linear scale correlation in the video images. Overall, the responses of V4 neurons depended on marginal statistics and correlation statistics to a greater extent than the responses of V1 neurons. We further showed that neurons with different selectivities to PS statistics were distributed unevenly within each area, and neighboring neuron pairs exhibited more similar selectivities to PS statistics than distant neuron pairs. Of particular note is that different imaging sites in V4 showed drastically different proportions of neurons tuned to spectral, marginal, or correlation statistics, suggesting that V4 contained discrete subregions with different selectivities to PS statistics.

### Encoding-model approach to studies of neuronal tuning

We exploited an encoding-model approach to analyze the effect of a large number of PS statistics and to minimize the overfitting problem by incorporating regularization. The prediction performance of the models varied greatly across neurons, but the distribution deviated from zero towards positive values (Fig. 3), indicating that the encoding model was able to capture the responses to the naturalistic videos in a significant subpopulation of V1 and V4 neurons. The prediction performance of the encoding models was higher for V1 neurons than for V4 neurons (Fig. 3b). The positive correlation between explainable variance and prediction performance (insets in Fig. 3b) suggests that the higher performance of the V1 models may be at least partly due to the more consistent responses of V1 neurons across trials (Fig. 2b).

The use of naturalistic videos as visual stimuli has both merits and demerits. On one hand, this approach allows us to characterize neuronal selectivities to marginal statistics that change dynamically during natural vision. We found that marginal statistics were crucial in determining the responses of a large population of neurons in V1 and V4 (Fig. 5a, b), and these responses were affected by many aspects of the luminance distribution, such as the mean, variances, skewness, and kurtosis (Fig. 6). This sensitivity to marginal statistics has been underestimated in previous studies using stimuli with equalized marginal statistics across images. We implemented PS-statistics filters as the initial filters in our models, because the ventral visual pathway areas process shape and texture that can be synthesized from PS-statistics-based computation (Portilla and Simoncelli 2000; Freeman and Simoncelli 2011). On the other hand, our PS-statistics-based models cannot capture responses to other visual cues in the videos, such as color and motion. Given the selectivities of V1 and V4 neurons to color (Komatsu 1998; Gegenfurtner 2003; Roe et al. 2012), it will be interesting to see if the addition of color detection filters to the models improves prediction performance. An encoding model that used a motion energy model (Ikezoe et al. 2018) achieved higher prediction performance in V1 (mean ± SD, 0.36 ± 0.15, *n* = 2146) but comparable performance in V4 (0.16 ± 0.12, *n* = 2492) compared to performances with the PS-statistics model in V1 (0.18 ± 0.13) and V4 (0.11 ± 0.11). This finding suggests that motion plays an important role in determining the responses of the V1 neurons in our sample, i.e., the V1 neurons in layer 2 and the uppermost tier of layer 3.

Our encoding models included only spatial image statistics and hence do not capture the large contribution of temporal factors to visual responses, which may co-vary with some of the PS statistics. This raises a concern whether neurons may be driven by a temporal factor co-varying with a static PS factor. We did find a moderate correlation (median: 0.24) in the spectral group of PS statistics (see “Materials and methods”). This is reasonable because both the spectral statistics and Gabor filters are in most cases strongly selective to orientation and spatial frequency. The non-spectral group of PS statistics (i.e., marginal and correlation statistics) exhibited a much weaker correlation with local flicker/motion energy (median: 0.094). Although the correlations between the PS statistics and flicker/motion energy were relatively weak, especially for the non-spectral group, their potential effect on our interpretation should be taken into account carefully. A straightforward, yet demanding, approach to this issue may be to test a neuron with a set of controlled, non-moving stimuli to determine the contribution of the PS statistics of interest. To achieve this, one may need to first perform experiments with the current method, and then test the same neuron with controlled, non-moving stimuli in additional experiments. We were unable to conduct this additional testing because the fluorescent calcium indicator we used, Cal-520 AM, faded away within a day. Genetically encoded calcium indicators such as GCaMPs may enable long-term recordings (Kimura et al. 2021) and allow us to perform the experiments above.

Both the explainable variance and prediction performance were rather low in this study. The adaptive stimulus sampling procedure customizes the stimulus set to the properties of the targeted individual neuron, thus making it possible to achieve higher prediction accuracy (Okazawa et al. 2015). Our encoding-model analysis does not include a stimulus optimization procedure for individual neurons, but instead is applicable to simultaneously recorded responses from multiple neurons. Using two-photon calcium imaging, we were able to characterize the selectivities of several tens to hundreds of neurons in one experiment, resulting in a database consisting of up to a few thousand neurons in total, a substantially larger number than previously examined. Thus, there is a trade-off between model accuracy, achieved with single-neuron recording and the adaptive stimulus sampling procedure, and larger database size, which can be realized using two-photon calcium imaging of multiple neurons with the encoding-model approach. The information derived from our large database can complement the results of previous studies.

### Tunings to PS statistics in V1 and V4

In V1 and V4, spectral statistics, marginal statistics, and linear scale correlation were major factors that explained the responses to the naturalistic videos (Fig. 5). Tuning to spectral statistics is consistent with the well-known selectivity of V1 and V4 neurons to the orientations and spatial frequencies of stimuli (e.g., for V1, Campbell et al. 1969; De Valois et al. 1982; Hubel and Wiesel 1962; Maffei and Fiorentini 1973; Movshon et al. 1978; for V4, Desimone and Schein 1987; Roe et al. 2012; Lu et al. 2018). We found that V4 neurons preferring spectral statistics tended also to be tuned to correlation statistics, including both linear and energy correlations between different scales, orientations, and positions (Fig. 5a). Correlation between different scales is useful for representing edges, correlation between different orientations is useful for representing curves and angles, and correlation between different positions is useful for representing repeated patterns, such as visual texture. V4 neurons thus have properties suitable to respond to complex features, such as angled or curved contours, sharp edges, and periodic patterns of visual images. Previous studies have shown that a substantial population of V4 neurons respond to curved contours and gratings (Gallant et al. 1996; Pasupathy and Connor 1999), corners and crosses (Hegdé and Van Essen 2007), visual textures (Okazawa et al. 2015, 2017), and complex shapes (Kobatake and Tanaka 1994; Carlson et al. 2011).

A variety of spectral information coded by different V1 neurons can converge into single V4 neurons in different ways. If a V4 neuron combines information of the same orientation from different positions, it will have a large receptive field and be categorized as cross-positional. If a V4 neuron combines information of different orientations from the same position, it will have a small receptive field and be categorized as cross-orientational. A previous study suggested that both types of neurons exist in V4 (Nandy et al. 2013). Furthermore, in V2, there is no correlation between receptive field size and strength of response modulation by naturalistic textures that contain correlation statistics (Freeman et al. 2013). Therefore, the tuning to correlation statistics in a larger population of V4 neurons is unlikely to simply reflect the larger sizes of their receptive fields compared to V1 neurons.

We showed that 37% of V1 neurons and 52% of V4 neurons were preferentially tuned to marginal statistics (Fig. 5a), whereas previous studies found only a small number of such neurons (< 10%; see Fig. 5C of Okazawa et al. 2017). The abundance of neurons tuned to marginal statistics was revealed using naturalistic videos as stimuli. The contents of clips and frames in these videos drastically differed, and no normalization was applied to compensate for large differences in the luminance histograms across clips and frames. The marginal statistics computed from the luminance histograms, therefore, dynamically changed along the time axis. In contrast, previous studies equalized the mean and variance of luminance between their stimuli (Okazawa et al. 2015, 2017). Our results indicate that marginal statistics, including skewness as well as the mean and variance of luminance, are important factors in determining the responses to natural scenes in both V1 and V4 (Fig. 6). Indeed, some V1 neurons selectively respond to the luminance level of uniform surfaces that lack spectral statistics (Maguire and Baizer 1982; Rossi et al. 1996; Kinoshita and Komatsu 2001). Visual features unrelated to spectral and correlation statistics are likely to be represented in V1 and further processed in V4.

Linear scale correlation underlies the representation of visual features produced by interactions across different spatial frequencies. For example, edge-like and line-like features are produced by the same orientation, but with different phase congruence across spatial frequencies. Selective responses to such phase congruence were found in V1 neurons in studies using compound gratings containing multiple spatial frequencies (Mechler et al. 2002). The neuronal tuning to linear scale correlation that we observed in V1 and V4 neurons might explain the selectivity to the spatial phase congruence.

Comparisons of fitting weights revealed that spectral statistics contributed more to neuronal responses in V1 than in V4 (Fig. 5b). In contrast, the contribution of marginal and correlation statistics was greater in V4 than in V1 (Fig. 5c). These results are consistent with those of previous studies supporting the gradual development of neural representations for higher order features along the ventral visual pathway (Freeman et al. 2013; Okazawa et al. 2017).

### Functional subregions in V4

In V1 and V4, the proportions of the preferred statistics depended on the imaging sites (Figs. 7, 8). In V1, the proportions of neurons tuned to spectral statistics and those tuned to marginal statistics varied gradually across sites, while the proportion of neurons tuned to correlation statistics remained consistently low (Fig. 8a, upper panel). The differential distributions of neurons with different stimulus selectivities were more prominent in V4 (Fig. 8a, lower panel); some sites contained a high proportion (50–70%) of neurons tuned to correlation statistics, whereas other sites contained a much lower proportion (10–30%) of such neurons, and a few sites consisted predominantly of neurons tuned to spectral statistics. Furthermore, nearby neurons within a single site tended to show more similar preferences for PS statistics than distant neurons (Fig. 9). These findings are further evidence for the existence of functional subregions within V4 as reported by studies using functional MRI and intrinsic signal optical imaging. Activation caused by color or orientation of stimulus gratings appears as separate spots in V4 (Conway et al. 2007; Tanigawa et al. 2010). The V4 sites with many neurons tuned to spectral statistics may be located in orientation-selective subregions. Furthermore, the V4 sites containing neurons tuned to correlation statistics may preferentially respond to visual texture (Okazawa et al. 2017). Although clustering of neurons with texture sensitivity has not been examined to date, Gallant et al. (1996) previously suggested clustering of neurons that respond to similar non-Cartesian (curved) gratings. A recent study using intrinsic signal optical imaging demonstrated that curvatures of different degrees were processed in sub-millimeter-sized modules in V4 (Hu et al. 2020). Single-neuron recording studies also suggest that neurons with sensitivities to binocular disparity and disparity-defined edges are locally clustered in V4 (Watanabe et al. 2002; Tanabe et al. 2005; Fang et al. 2019). The combined application of macroscopic mapping using fMRI or intrinsic signal optical imaging with microscopic mapping using two-photon calcium imaging may shed light on the overall organization and finer details of these functional subregions of V4.

## Data Availability

The data sets for figures are available upon reasonable request to the corresponding author. Visual stimuli (https://crcns.org/data-sets/vc/vim-2/abpit-vim-2) and part of the fluorescence data (https://ai-data.nict.go.jp/dataset/detail/?id=35) used in this study can also be found online.
